# Supplementary material for: Metabolite-Responsive Control of Transcription by Phase Separation-Based Synthetic Organelles
Source: ACS Synth Biol. 2025 Feb 15;14(3):711–8. doi: 10.1021/acssynbio.4c00633 (PMC11934134; doi:10.1021/acssynbio.4c00633)
Supplement: Supplementary file 1 — sb4c00633_si_001.pdf [file sb4c00633_si_001.pdf]

Metabolite-responsive Control of Transcription by Phase Separation-based Synthetic Organelles

Carolina Jerez-Longres[1,3] and Wilfried Weber[1,2,3]*

[1] INM – Leibniz Institute for New Materials, Campus D2 2, 66123 Saarbrücken, Germany

[2] Saarland University, Department of Materials Science and Engineering, 66123 Saarbrücken, Germany

[3] Signalling research Centers BIOSS and CIBSS, Faculty of Biology, and SGBM - Spemann Graduate School of Biology and Medicine, University of Freiburg, Schänzlestrasse 18, 79104 Freiburg, Germany

*To whom correspondence should be addressed: wilfried.weber@leibniz-inm.de

Supplementary information

Table S1. Plasmids generated in this study

Name	Description	Sequence 5' -> 3'
**pCJL236**	T_7 promoter-MBP-3CS-PdhR-FUS_N<br><br>Backbone: pRG001 [34]	…GCCCTTCACCTAATACGACTCACTATAGGGGAATTGTGAGCGGATAACA<br>ATTCCCCTCTAGAAATAATTTTGTTTAACTTTAAGAAGGAATTCAGGAGCC<br>CTTCACCATGAAAATCGAAGAAGGTAAACTGGTAATCTGGATTAACGGCG<br>ATAAAGGCTATAACGGTCTCGCTGAAGTCGGTAAGAAATTCGAGAAAGAT<br>ACCGGAATTAAAGTCACCGTTGAGCATCCGGATAAACTGGAAGAGAAATT<br>CCCACAGGTTGCGGCAACTGGCGATGGCCCTGACATTATCTTCTGGGCAC<br>ACGACCGCTTTGGTGGCTACGCTCAATCTGGCCTGTTGGCTGAAATCACCC<br>CGGACAAAGCGTTCCAGGACAAGCTGTATCCGTTTACCTGGGATGCCGTA<br>CGTTACAACGGCAAGCTGATTGCTTACCCGATCGCTGTTGAAGCGTTATCG<br>CTGATTTATAACAAAGATCTGCTGCCGAACCCGCCAAAAACCTGGGAAGA<br>GATCCCGGCGCTGGATAAAGAACTGAAAGCGAAAGGTAAGAGCGCGCTG<br>ATGTTCAACCTGCAAGAACCGTACTTCACCTGGCCGCTGATTGCTGCTGAC<br>GGGGGTTATGCGTTCAAGTATGAAAACGGCAAGTACGACATTAAAGACGT<br>GGGCGTGGATAACGCTGGCGCGAAAGCGGGTCTGACCTTCCTGGTTGACC<br>TGATTAAAAACAAACACATGAATGCAGACACCGATTACTCCATCGCAGAA<br>GCTGCCTTTAATAAAGGCGAAACAGCGATGACCATCAACGGCCCGTGGGC<br>ATGGTCCAACATCGACACCAGCAAAGTGAATTATGGTGTAACGGTACTGC<br>CGACCTTCAAGGGTCAACCATCCAAACCGTTCGTTGGCGTGCTGAGCGCA<br>GGTATTAACGCCGCCAGTCCGAACAAAGAGCTGGCAAAAGAGTTCCTCGA<br>AAACTATCTGCTGACTGATGAAGGTCTGGAAGCGGTTAATAAAGACAAAC<br>CGCTGGGTGCCGTAGCGCTGAAGTCTTACGAGGAAGAGTTGGCGAAAGA<br>TCCACGTATTGCCGCCACTATGGAAAACGCCCAGAAAGGTGAAATCATGC<br>CGAACATCCCGCAGATGTCCGCTTTCTGGTATGCCGTGCGTACTGCGGTGA<br>TCAACGCCGCCAGCGGTCGTCAGACTGTCGATGAAGCCCTGAAAGACGCG<br>CAGACTAATTCGAGCTCGAACAACAACAACAATAACAATAACAACAACCTC<br>GGGATCGAGGGAAGGGGTGGAGGCGGATCGCTGGAAGTTCTGTTCCAG

		GGGCCCATGGCCTACAGCAAAATCCGCCAACCAAAACTCTCCGATGTGATT<br>GAGCAGCAACTGGAGTTTTTGATCCTCGAAGGCACTCTCCGCCCGGGCGA<br>AAAACTCCCACCGGAACGCGAACTGGCAAAACAGTTTGACGTCTCCCGTC<br>CCTCCTTGCGTGAGGCGATTCAACGTCTCGAAGCGAAGGGCTTGTTGCTTC<br>GTCGCCAGGGTGGCGGCACTTTTGTCCAGAGCAGCCTATGGCAAAGCTTC<br>AGCGATCCGCTGGTGGAGCTGCTCTCCGACCATCCTGAGTCACAGTATGA<br>CTTGCTCGAAACACGACACGCCCTGGAAGGTATCGCCGCTTATTACGCCGC<br>GCTGCGTAGTACCGATGAAGACAAGGAACGCATCCGTGAACTCCACCACG<br>CCATAGAGCTGGCGCAGCAGTCTGGCGATCTGGACGCGGAATCAAACGCC<br>GTACTCCAGTATCAGATTGCCGTCACCGAAGCGGCCCACAATGTGGTTCTG<br>CTTCATCTGCTAAGGTGTATGGAGCCGATGTTGGCCCAGAATGTCCGCCA<br>GAACTTCGAATTGCTCTATTCGCGTCGCGAGATGCTGCCGCTGGTGAGTA<br>GTCACCGCACCCGCATATTTGAAGCGATTATGGCCGGTAAGCCGGAAGAA<br>GCGCGCGAAGCATCGCATCGCCATCTGGCCTTTATCGAAGAAATTTTGCTC<br>GACAGAAGTCGTGAAGAGAGCCGCCGTGAGCGTTCTCTGCGTCGTCTGGA<br>GCAACGAAAGAATAAGGGCGAGCTCAATTCGAAGCTTGAAGGTAAGCCT<br>ATCCCTAACCCTCTCCTCGGTCTCGATTCTACGCGTACCGGTGGTGGAGGA<br>ATGGCCTCAAACGATTATACCCAACAAGCAACCCAAAGCTATGGGGCCTA<br>CCCCACCCAGCCCGGGCAGGGCTATTCCCAGCAGAGCAGTCAGCCCTACG<br>GACAGCAGAGTTACAGTGGTTATAGCCAGTCCACGGACACTTCAGGCTAT<br>GGCCAGAGCAGCTATTCTTCTTATGGCCAGAGCCAGAACACAGGCTATGG<br>AACTCAGTCAACTCCCCAGGGATATGGCTCGACTGGCGGCTATGGCAGTA<br>GCCAGAGCTCCCAATCGTCTTACGGGCAGCAGTCCTCCTATCCTGGCTATG<br>GCCAGCAGCCAGCTCCCAGCAGCACCTCGGGAAGTTACGGTAGCAGTTCT<br>CAGAGCAGCAGCTATGGGCAGCCCCAGAGTGGGAGCTACAGCCAGCAGC<br>CTAGCTATGGTGGACAGCAGCAAAGCTATGGACAGCAGCAAAGCTATAAT<br>CCCCCTCAGGGCTATGGACAGCAGAACCAGTACAACAGCAGCAGTGGTG<br>GTGGAGGTGGAGGTGGAGGTGGAGGTAACTATGGCCAAGATCAATCCTC<br>CATGAGTAGTGGTGGTGGCAGTGGTGGCGGTTATGGCAATCAAGACCAG<br>AGTGGTGGAGGTGGCAGCGGTGGCTATGGACAGCAGGACCGTGGACATC<br>ATCACCATCACCATTGAGTTTGAT…
**pCJL241**	*pdhO*-spacer-<br>T_7 promoter-<br>SdBroccoli<br><br>Backbone:<br>SdB [38]	…AGCGTCTAGAGACATGAAATTGGTAAGACCAATTGACTTCGGGCTAGAG<br>AGAGCACTAACCCATCAACCTGTACGGGAACATTCTATATCGTTCTCGGAC<br>GGACAGATTACTAGAGTGCCGCTTTCAGCCCCTCTGTCGTCGCCGACGTCT<br>GTAATATGGCGGCTAGACGATCCCGCGAAATTAATACGACTCACTATAGG<br>AGGGAGACGGTCGGGTCCATCTGAGACGGTCGGGTCCAGATATTCGTATC<br>TGTCGAGTAGAGTGTGGGCTCAGATGTCGAGTAGAGTGTGGGCTCCCTCT<br>AGCATAAC…
**pCJL244**	*pdhO*$_6$-spacer-<br>T_7 promoter-<br>SdBroccoli<br><br>Backbone:<br>SdB [38]	…AGCGTCTAGAGACATGAAATTGGTAAGACCAATTGACTTCGGGCTAGAG<br>ACATGAAATTGGTAAGACCAATTGACTTCGGGCTAGAGACATGAAATTGG<br>TAAGACCAATTGACTTCGGGCTAGAGAGAGCACTAACCCGCTAGAGACAT<br>GAAATTGGTAAGACCAATTGACTTCGGGCTAGAGACATGAAATTGGTAAG<br>ACCAATTGACTTCGGGCTAGAGACATGAAATTGGTAAGACCAATTGACTTC<br>GGGCTAGAGAGAGCACTAACCCATCAACCTGTACGGGAACATTCTATATC<br>GTTCTCGGACGGACAGATTACTAGAGTGCCGCTTTCAGCCCCTCTGTCGTC<br>GCCGACGTCTGTAATATGGCGGCTAGACGATCCCGCGAAATTAATACGAC<br>TCACTATAGGAGGGAGACGGTCGGGTCCATCTGAGACGGTCGGGTCCAG<br>ATATTCGTATCTGTCGAGTAGAGTGTGGGCTCAGATGTCGAGTAGAGTGT<br>GGGCTCCCTCTAGCATAAC…

**pCJL240**	$pdhO^{*}_{6}$-spacer-T_7 promoter-SdBroccoli<br><br>Backbone: SdB [38]	…ATCGTCTAGCAATTGGTCTTACCAATTTCTAGCAATTGGTCTTACCAATTT<br>CTAGCAATTGGTCTTACCAATTTCTAGCATTTCGCGGGATCGTCTAGCAATT<br>GGTCTTACCAATTTCTAGCAATTGGTCTTACCAATTTCTAGCAATTGGTCTT<br>ACCAATTTCTAGAGAGAGCACTAACCCATCAACCTGTACGGGAACATTCTA<br>TATCGTTCTCGGACGGACAGATTACTAGAGTGCCGCTTTCAGCCCCTCTGT<br>CGTCGCCGACGTCTGTAATATGGCGGCTAGACGATCCCGCGAAATTAATA<br>CGACTCACTATAGGAGGGAGACGGTCGGGTCCATCTGAGACGGTCGGGT<br>CCAGATATTCGTATCTGTCGAGTAGAGTGTGGGCTCAGATGTCGAGTAGA<br>GTGTGGGCTCCCTCTAGCATAAC…
**pNS204**	T_7 promoter-MBP-3CS-FUS_N<br><br>Backbone: pHJW288 (unpublished)	…CCCGCGAAATTAATACGACTCACTATAGGGAGACCACAACGGTTTCCCT<br>CTAGAAATAATTTTGTTTAACTTTAAGAAGGAGATATACATATGAAAATCG<br>AAGAAGGTAAACTGGTAATCTGGATTAACGGCGATAAAGGCTATAACGGT<br>CTCGCTGAAGTCGGTAAGAAATTCGAGAAAGATACCGGAATTAAAGTCAC<br>CGTTGAGCATCCGGATAAACTGGAAGAGAAATTCCCACAGGTTGCGGCAA<br>CTGGCGATGGCCCTGACATTATCTTCTGGGCACACGACCGCTTTGGTGGCT<br>ACGCTCAATCTGGCCTGTTGGCTGAAATCACCCCGGACAAAGCGTTCCAG<br>GACAAGCTGTATCCGTTTACCTGGGATGCCGTACGTTACAACGGCAAGCT<br>GATTGCTTACCCGATCGCTGTTGAAGCGTTATCGCTGATTTATAACAAAGA<br>TCTGCTGCCGAACCCGCCAAAAACCTGGGAAGAGATCCCGGCGCTGGATA<br>AAGAACTGAAAGCGAAAGGTAAGAGCGCGCTGATGTTCAACCTGCAAGA<br>ACCGTACTTCACCTGGCCGCTGATTGCTGCTGACGGGGGTTATGCGTTCAA<br>GTATGAAAACGGCAAGTACGACATTAAAGACGTGGGCGTGGATAACGCT<br>GGCGCGAAAGCGGGTCTGACCTTCCTGGTTGACCTGATTAAAAACAAACA<br>CATGAATGCAGACACCGATTACTCCATCGCAGAAGCTGCCTTTAATAAAGG<br>CGAAACAGCGATGACCATCAACGGCCCGTGGGCATGGTCCAACATCGACA<br>CCAGCAAAGTGAATTATGGTGTAACGGTACTGCCGACCTTCAAGGGTCAA<br>CCATCCAAACCGTTCGTTGGCGTGCTGAGCGCAGGTATTAACGCCGCCAG<br>TCCGAACAAAGAGCTGGCAAAAGAGTTCCTCGAAAACTATCTGCTGACTG<br>ATGAAGGTCTGGAAGCGGTTAATAAAGACAAACCGCTGGGTGCCGTAGC<br>GCTGAAGTCTTACGAGGAAGAGTTGGCGAAAGATCCACGTATTGCCGCCA<br>CTATGGAAAACGCCCAGAAAGGTGAAATCATGCCGAACATCCCGCAGATG<br>TCCGCTTTCTGGTATGCCGTGCGTACTGCGGTGATCAACGCCGCCAGCGGT<br>CGTCAGACTGTCGATGAAGCCCTGAAAGACGCGCAGACTAATTCGAGCTC<br>GAACAACAACAACAATAACAATAACAACAACCTCGGGATCGAGGGAAGG<br>GGTGGAGGCGGATCGCTGGAAGTTCTGTTCCAGGGGCCCATGGACAGGT<br>GCGGCCACCACCACCACCACCACATGGCCTCAAACGATTATACCCAACAAG<br>CAACCCAAAGCTATGGGGCCTACCCCACCCAGCCCGGGCAGGGCTATTCC<br>CAGCAGAGCAGTCAGCCCTACGGACAGCAGAGTTACAGTGGTTATAGCCA<br>GTCCACGGACACTTCAGGCTATGGCCAGAGCAGCTATTCTTCTTATGGCCA<br>GAGCCAGAACACAGGCTATGGAACTCAGTCAACTCCCCAGGGATATGGCT<br>CGACTGGCGGCTATGGCAGTAGCCAGAGCTCCCAATCGTCTTACGGGCAG<br>CAGTCCTCCTACCCTGGCTATGGCCAGCAGCCAGCTCCCAGCAGCACCTCG<br>GGAAGTTACGGTAGCAGTTCTCAGAGCAGCAGCTATGGGCAGCCCCAGA<br>GTGGGAGCTACAGCCAACAGCCTAGCTATGGTGGACAGCAGCAATCTTAC<br>GGTCAACAACAGAGCTATAATCCCCCTCAGGGCTATGGACAGCAGAACCA<br>GTACAACAGCAGCAGTGGTGGTGGAGGTGGAGGTGGAGGTGGAGGTAA<br>CTATGGCCAAGATCAATCCTCCATGAGTAGTGGTGGTGGCAGTGGTGGCG<br>GTTATGGCAATCAAGACCAGAGTGGTGGAGGTGGCAGCGGTGGCTATGG<br>ACAGCAGGACCGTGGATAAAAGCTTG…

**pCJL222**	T_7 promoter-MBP-3CS-PdhR-FUS_N-avitag<br>Backbone: pRG001 [34]	…GCCCTTCACCTAATACGACTCACTATAGGGGAATTGTGAGCGGATAACA<br>ATTCCCCTCTAGAAATAATTTTGTTTAACTTTAAGAAGGAATTCAGGAGCC<br>CTTCACCATGAAAATCGAAGAAGGTAAACTGGTAATCTGGATTAACGGCG<br>ATAAAGGCTATAACGGTCTCGCTGAAGTCGGTAAGAAATTCGAGAAAGAT<br>ACCGGAATTAAAGTCACCGTTGAGCATCCGGATAAACTGGAAGAGAAATT<br>CCCACAGGTTGCGGCAACTGGCGATGGCCCTGACATTATCTTCTGGGCAC<br>ACGACCGCTTTGGTGGCTACGCTCAATCTGGCCTGTTGGCTGAAATCACCC<br>CGGACAAAGCGTTCCAGGACAAGCTGTATCCGTTTACCTGGGATGCCGTA<br>CGTTACAACGGCAAGCTGATTGCTTACCCGATCGCTGTTGAAGCGTTATCG<br>CTGATTTATAACAAAGATCTGCTGCCGAACCCGCCAAAAACCTGGGAAGA<br>GATCCCGGCGCTGGATAAAGAACTGAAAGCGAAAGGTAAGAGCGCGCTG<br>ATGTTCAACCTGCAAGAACCGTACTTCACCTGGCCGCTGATTGCTGCTGAC<br>GGGGGTTATGCGTTCAAGTATGAAAACGGCAAGTACGACATTAAAGACGT<br>GGGCGTGGATAACGCTGGCGCGAAAGCGGGTCTGACCTTCCTGGTTGACC<br>TGATTAAAAACAAACACATGAATGCAGACACCGATTACTCCATCGCAGAA<br>GCTGCCTTTAATAAAGGCGAAACAGCGATGACCATCAACGGCCCGTGGGC<br>ATGGTCCAACATCGACACCAGCAAAGTGAATTATGGTGTAACGGTACTGC<br>CGACCTTCAAGGGTCAACCATCCAAACCGTTCGTTGGCGTGCTGAGCGCA<br>GGTATTAACGCCGCCAGTCCGAACAAAGAGCTGGCAAAAGAGTTCCTCGA<br>AAACTATCTGCTGACTGATGAAGGTCTGGAAGCGGTTAATAAAGACAAAC<br>CGCTGGGTGCCGTAGCGCTGAAGTCTTACGAGGAAGAGTTGGCGAAAGA<br>TCCACGTATTGCCGCCACTATGGAAAACGCCCAGAAAGGTGAAATCATGC<br>CGAACATCCCGCAGATGTCCGCTTTCTGGTATGCCGTGCGTACTGCGGTGA<br>TCAACGCCGCCAGCGGTCGTCAGACTGTCGATGAAGCCCTGAAAGACGCG<br>CAGACTAATTCGAGCTCGAACAACAACAACAATAACAATAACAACAACCTC<br>GGGATCGAGGGAAGGGGTGGAGGCGGATCGCTGGAAGTTCTGTTCCAG<br>GGGCCCATGGCCTACAGCAAAATCCGCCAACCAAAACTCTCCGATGTGATT<br>GAGCAGCAACTGGAGTTTTTGATCCTCGAAGGCACTCTCCGCCCGGGCGA<br>AAAACTCCCACCGGAACGCGAACTGGCAAAACAGTTTGACGTCTCCCGTC<br>CCTCCTTGCGTGAGGCGATTCAACGTCTCGAAGCGAAGGGCTTGTTGCTTC<br>GTCGCCAGGGTGGCGGCACTTTTGTCCAGAGCAGCCTATGGCAAAGCTTC<br>AGCGATCCGCTGGTGGAGCTGCTCTCCGACCATCCTGAGTCACAGTATGA<br>CTTGCTCGAAACACGACACGCCCTGGAAGGTATCGCCGCTTATTACGCCGC<br>GCTGCGTAGTACCGATGAAGACAAGGAACGCATCCGTGAACTCCACCACG<br>CCATAGAGCTGGCGCAGCAGTCTGGCGATCTGGACGCGGAATCAAACGCC<br>GTACTCCAGTATCAGATTGCCGTCACCGAAGCGGCCCACAATGTGGTTCTG<br>CTTCATCTGCTAAGGTGTATGGAGCCGATGTTGGCCCAGAATGTCCGCCA<br>GAACTTCGAATTGCTCTATTCGCGTCGCGAGATGCTGCCGCTGGTGAGTA<br>GTCACCGCACCCGCATATTTGAAGCGATTATGGCCGGTAAGCCGGAAGAA<br>GCGCGCGAAGCATCGCATCGCCATCTGGCCTTTATCGAAGAAATTTTGCTC<br>GACAGAAGTCGTGAAGAGAGCCGCCGTGAGCGTTCTCTGCGTCGTCTGGA<br>GCAACGAAAGAATAAGGGCGAGCTCAATTCGAAGCTTGAAGGTAAGCCT<br>ATCCCTAACCCTCTCCTCGGTCTCGATTCTACGCGTACCGGTGGTGGAGGA<br>ATGGCCTCAAACGATTATACCCAACAAGCAACCCAAAGCTATGGGGCCTA<br>CCCCACCCAGCCCGGGCAGGGCTATTCCCAGCAGAGCAGTCAGCCCTACG<br>GACAGCAGAGTTACAGTGGTTATAGCCAGTCCACGGACACTTCAGGCTAT<br>GGCCAGAGCAGCTATTCTTCTTATGGCCAGAGCCAGAACACAGGCTATGG<br>AACTCAGTCAACTCCCCAGGGATATGGCTCGACTGGCGGCTATGGCAGTA<br>GCCAGAGCTCCCAATCGTCTTACGGGCAGCAGTCCTCCTATCCTGGCTATG

GCCAGCAGCCAGCTCCCAGCAGCACCTCGGGAAGTTACGGTAGCAGTTCT
CAGAGCAGCAGCTATGGGCAGCCCCAGAGTGGGAGCTACAGCCAGCAGC
CTAGCTATGGTGGACAGCAGCAAAGCTATGGACAGCAGCAAAGCTATAAT
CCCCCTCAGGGCTATGGACAGCAGAACCAGTACAACAGCAGCAGTGGTG
GTGGAGGTGGAGGTGGAGGTGGAGGTAACTATGGCCAAGATCAATCCTC
CATGAGTAGTGGTGGTGGCAGTGGTGGCGGTTATGGCAATCAAGACCAG
AGTGGTGGAGGTGGCAGCGGTGGCTATGGACAGCAGGACCGTGGAGGT
GGAGGAGGCCTGAACGACATCTTCGAGGCTCAGAAAATCGAATGGCACG
AACATCATCACCATCACCATTGAGTTTGAT…

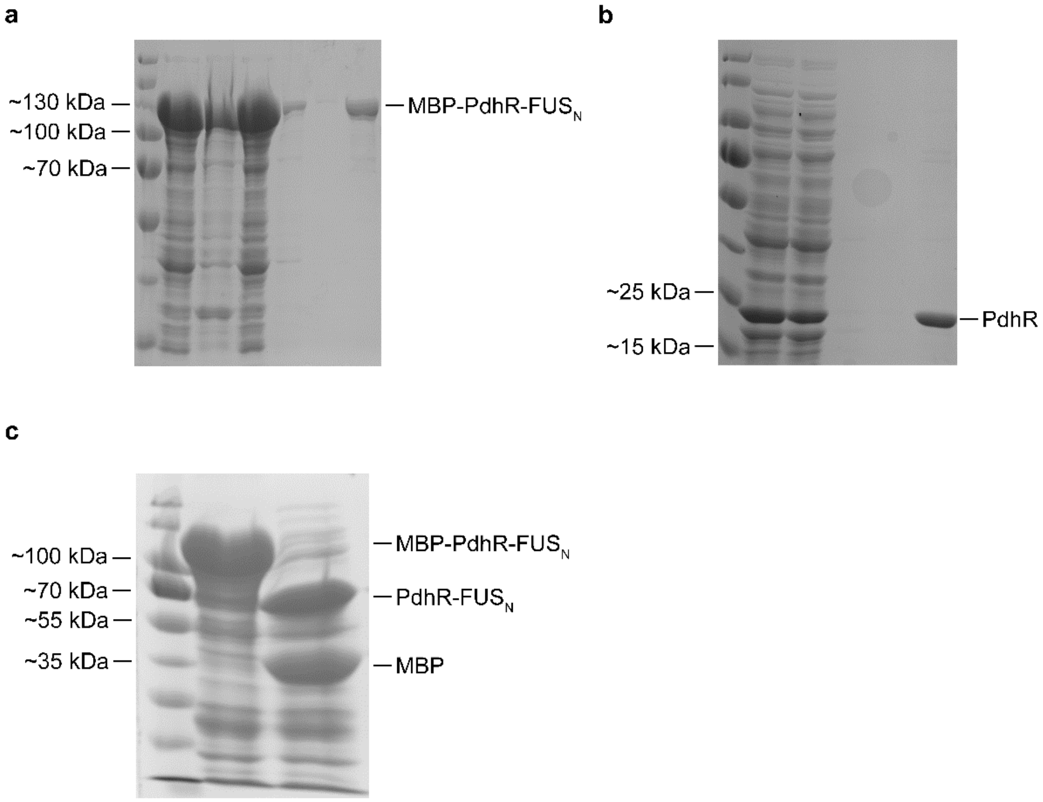

Figure S1. Purification and cleavage of PdhR variants. **(a)** Purification of MBP-PdhR-FUS$_N$. MBP-PdhR-FUS$_N$ encoded on plasmid pCJL236 was produced in *E. coli* and purified by immobilized metal affinity chromatography (IMAC). Samples from the different purification phases were analyzed by SDS-PAGE and Coomassie staining. Order of samples is (from left to right): protein size marker, clarified lysate, lysate pellet, flowthrough, first wash, second wash, eluate. **(b)** Purification of PdhR. PdhR encoded on plasmid pRG001 was produced and purified as described in (a). Order of samples is (from left to right): protein size marker, clarified lysate, flowthrough, first wash, second wash, eluate. **(c)** Digestion of MBP-PdhR-FUS$_N$ with 3C protease. MBP-PdhR-FUS$_N$ (70 µM) was incubated with 2.7 µM 3C protease and incubated for 24 h at RT. Samples before (middle lane) and after (right lane) digestion were analysed by SDS-PAGE and Coomassie staining. Right lane, protein size marker.

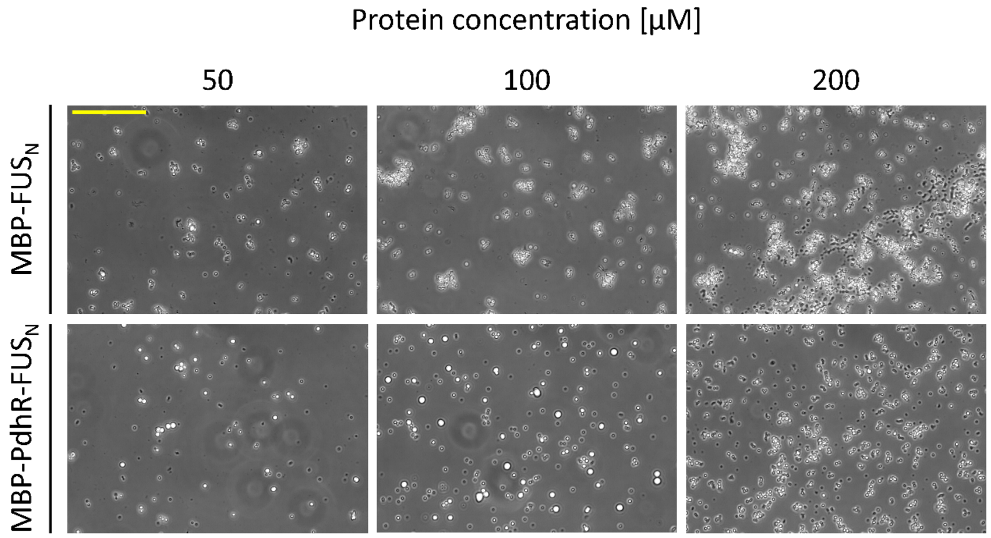

Figure S2. Comparison between the phase separation behaviour of FUS_N and PdhR-FUS_N. An MBP-FUS_N and an MBP-PdhR-FUS_N fusion proteins were produced from the pNS204 and pCJL222 plasmids, respectively (see **Table S1**), and incubated over night with 3C protease in a 50:1 (FUS_N construct : 3C protease) molar ratio. Finally, the samples were observed under a widefield microscope with phase contrast. Scale bar = 100 µm.

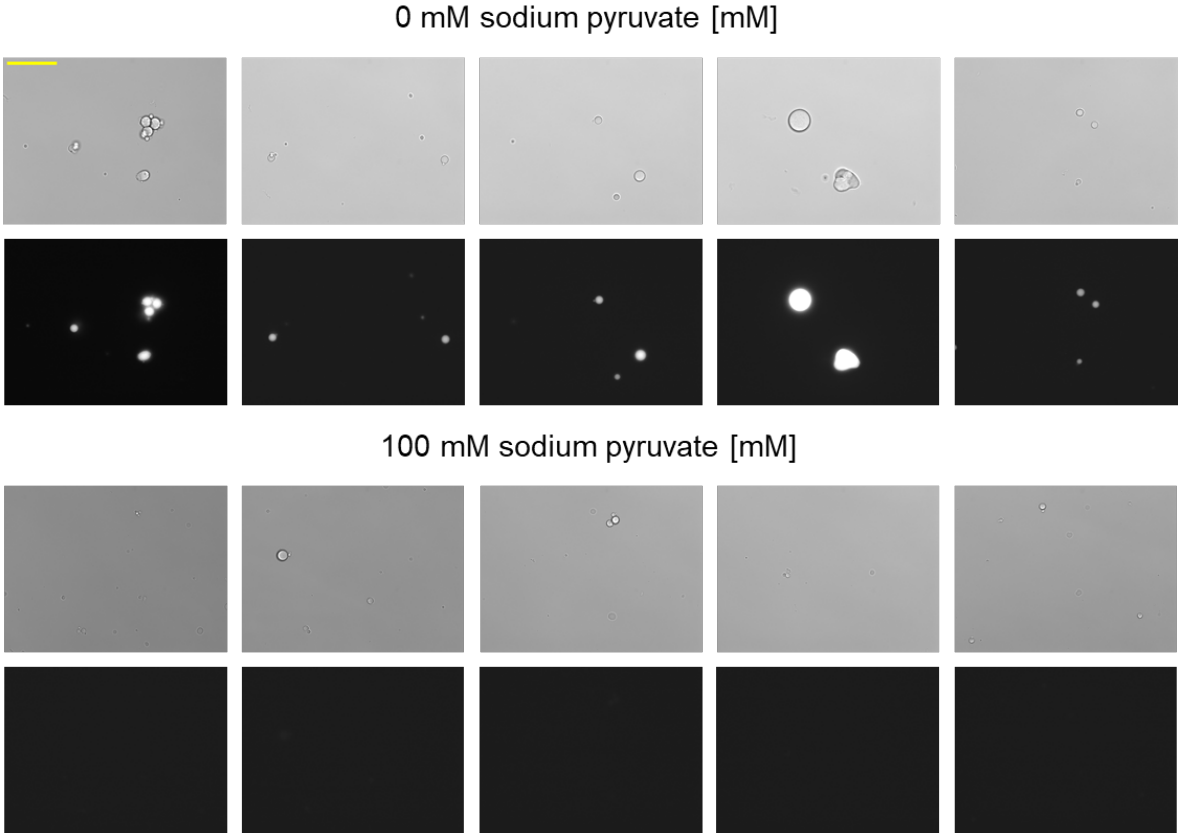

Figure S3. Pyruvate-responsive recruitment of *pdhO*-containing DNA into PdhR-based coacervates. 20 µM MBP-PdhR-FUS_N was mixed with 25 nM linear DNA encoding *pdhO*-T_7-SdBroccoli (pCJL241) for 30 min in the presence or absence of 100 mM pyruvate. 3C protease was added over night to remove the MBP solubility tag and to induce coacervate formation. DNA was visualized by DAPI staining and the samples were analysed by differential interference contrast (upper panels) and fluorescence microscopy (lower panels). Scale bar = 50 µm.

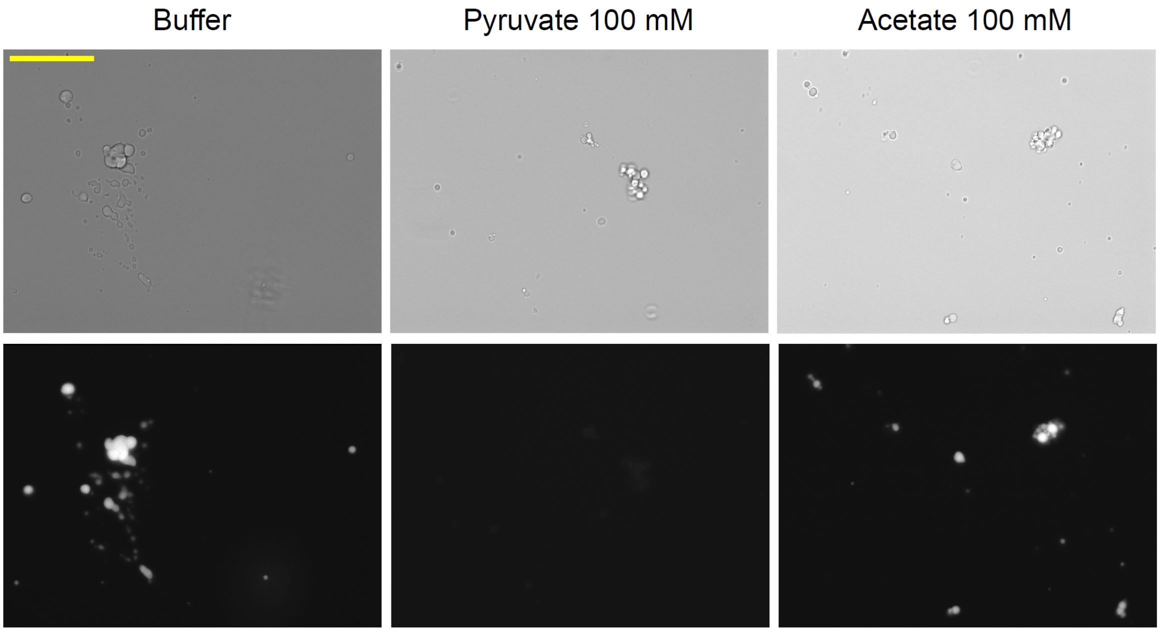

Figure S4. Effect of pyruvate on PdhR-FUS$_N$ and DNA condensates. The MBP-PdhR-FUS$_N$ protein and the pCJL241 DNA molecule were allowed to bind for 30 min at 37°C, and then incubated over night with 3C protease to cleave the MBP tag and induce condensate formation. Subsequently, 100 mM sodium-pyruvate or sodium-acetate were added, and the condensates were incubated for another 6 h. Finally, the samples were stained with DAPI and observed under DIC (upper panels) and fluorescence microscopy (lower panels). Scale bar = 50 µm.

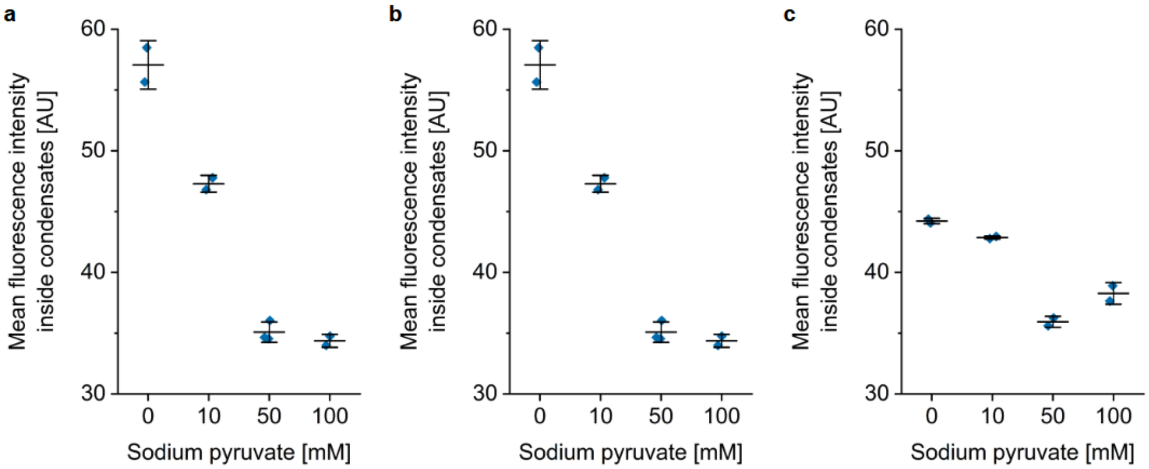

Figure S5. Quantification of the mean fluorescence intensity in regions delimitated by condensates at increasing pyruvate concentrations of the samples shown in **Figure 4**. Additional images used for quantification are not shown. Each dot represents the mean fluorescence intensity of all condensate-delimited regions in an individual image. Error bars represent the standard deviation. **(a)** pCJL241. **(b)** pCJL244. **(c)** pCJL240.

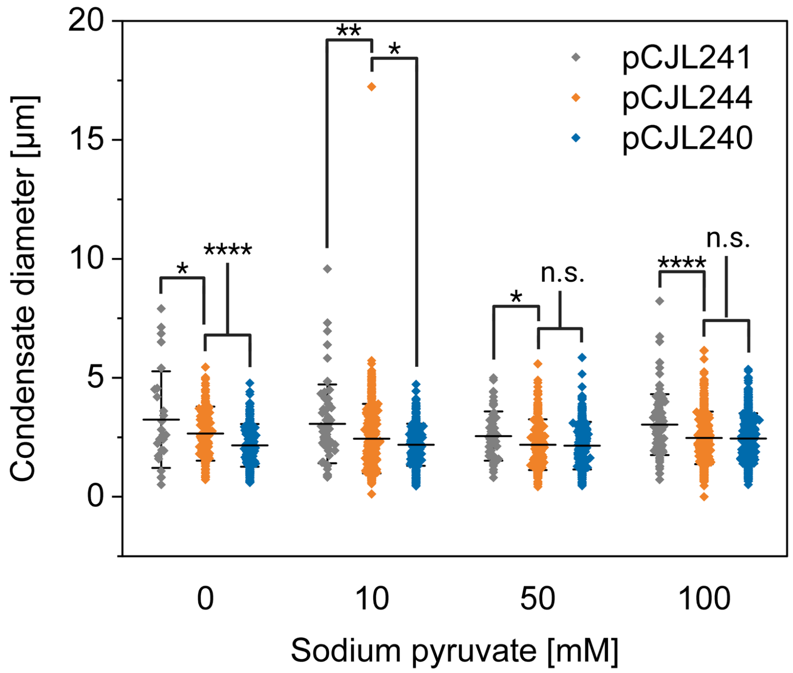

Figure S6. Diameters of individual condensates in the images shown in **Figure 4**. Error bars represent standard deviation. The asterisks indicate statistical significance with with a p-value of <0.05 (*), <0.01 (**), <0.0001 (****); n.s. = not significant.

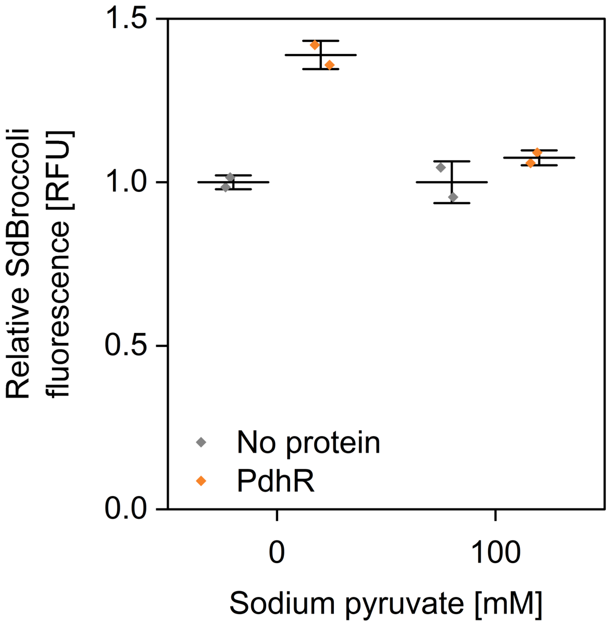

Figure S7. Effect of PdhR binding on SdBroccoli transcription in the presence or absence of 100 mM pyruvate. Results were normalized to the signal of the samples without protein with the same sodium pyruvate concentration. Error bars represent standard deviation.

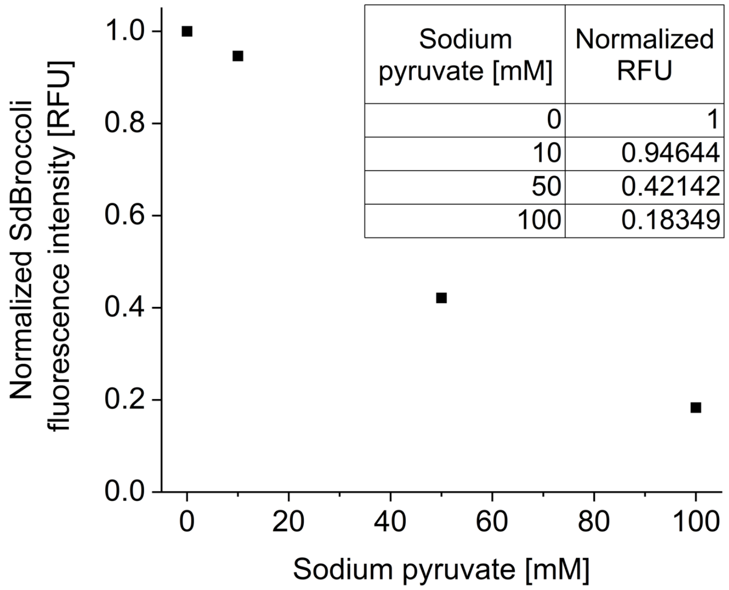

Sodium pyruvate [mM]	Normalized RFU
0	1
10	0.94644
50	0.42142
100	0.18349

Figure S8. SdBroccoli fluorescence of transcription reactions with pCJL241 and increasing sodium pyruvate concentrations. The results are normalized by the value of the sample with the highest signal (at 0 mM pyruvate). The transcription reactions were performed for 2 h at 37 °C and incubated with DFHBI-1T for an additional 30 min.

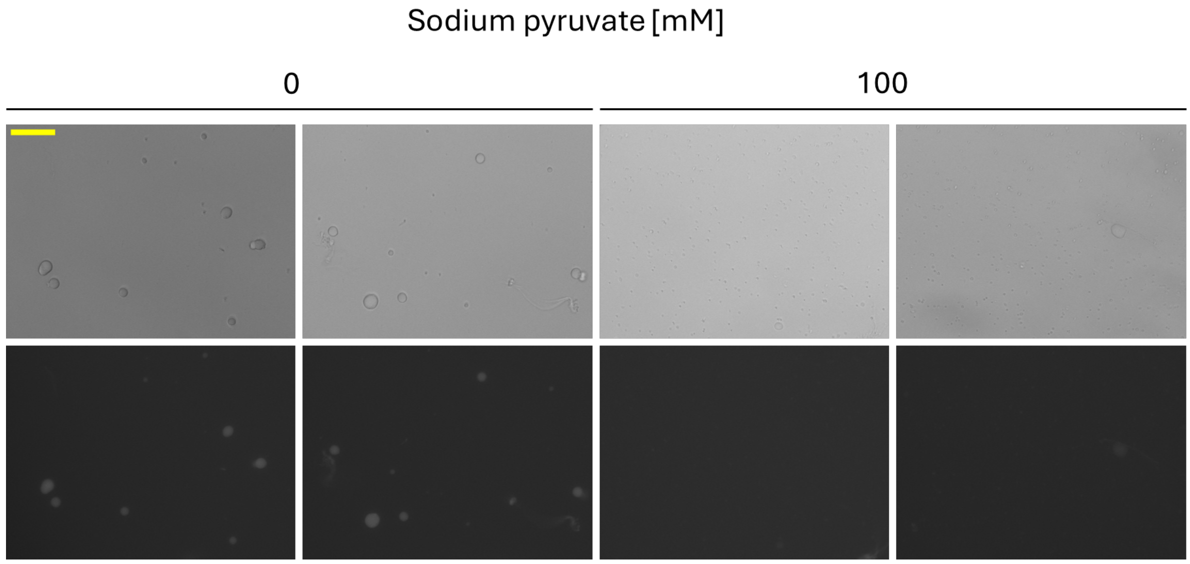

Figure S9. Localization of the SdBroccoli aptamer after transcription and addition of DFHBI-1T. Upper panels: DIC. Lower panels: SdBroccoli fluorescence. Scale bar = 50 µm.
